# Annual Address before the Dental Society of New York

**Published:** 1870-09

**Authors:** B. T. Whitney

**Affiliations:** Albany


Annual Address, Before the Dental Society of New York. By B. T. Whitney, D. D. S. Delivered in the Assembly Chamber, at Albany, June 29, 1870.

In compliance with the by-law requiring the President to deliver an annual address to this Society, I am before you this evening to discharge that duty. From the period in the history of Dentistry, the character of this organization, and the favorable circumstances under which we are together at this time, I have thought best to speak of the rise and progress of the profession, and its present status, especially in America, as more fitting than to write an essay on some particular branch of Dental science. Names and dates, like facts and figures, form but a dull subject; but I shall pass over them as briefly as may be consistent with what I wish to present most prominently: the influence for good of associated action, of society organizations.

To form any just estimate of one's present position or onward progress it is always necessary to look back upon the track over which we have passed, to take a retrospective view; without doing so we can not know of our advancement, appreciate improvements, or judge of the future.

The first Dentist in America, that I have been able to find any account of, was Isaac Greenwood; who was practicing in Boston in the year 1760. How, or of whom he obtained his knowledge, does not appear. His two sons, Clark and John, learning the art, settled in New York: the former in 1778, and the latter in 1783. They were probably the first American Dentists, and could not have had very large advantages for acquiring knowledge, yet they attained considerable skill and reputation. The latter made several sets of teeth for General Washington, which seem to have been well and successfully fitted, judging from Washington's expression of thanks. A Mr. Robert Wooffendale, from England, was the next after the elder Greenwood. He came to New York in Obtober, 1766, and divided his time between that city and Philadelphia for about eighteen months, when he returned to London. He came back in 1795, but soon retired to a farm in New Jersey, where he died in 1828, at the age of 87 years. His son John succeeded him in his practice in New York.

The next was a Frenchman, named LaMar, who came over in the French army with LaFayette, during the revolutionary war, and soon after its close an Englishman named

Whitlock. But they left no record of having been possessed of any great amount of knowledge or skill.

Next, we find the name of James Gardette: who was thoroughly educated for the French Navy, and as part of that education, was instructed in the dental art by M. Faudinier, a Dentist of high standing in Paris. He came to America with a naval detachment of LaFayette's army in January, 1788: bringing with him the best works extant on dentistry, and a complete set of instruments. After a few months he resigned his commission in the navy, and commenced the practice of dentistry in Boston; spending a portion of his time in Newport and other military stations. In 1783 he went to New York, and in the summer of 1784 permanently located in Philadelphia, where he remained in active practice until 1829, a period of forty-six years, when he returned to his native France, and died at Bordeaux in August, 1831, at the age of 75.

Though unaccustomed to the pen, he accomplished more, perhaps, than any one of his predecessors had done, in advancing the art of dentistry. He was the first to substitute gold clasps for ligatures of wire or thread in sustaining artificial teeth; thus enabling the patient to remove and cleanse them without the aid of a dentist. He was the first to support an artificial set of teeth by atmospheric pressure; which he did as early as the year 1800, though the demonstration of the principle, now so universally adopted, and so simple and effectual, was like many other important discoveries, the result of an accident. Artificial dentures were then made by carving them from blocks of ivory, or the tooth of the hippopotamus; which at best could secure but an approximate fit to the roof of the mouth and alveolar ridge. He was the first to use gold plates as a base for teeth, in place of the clumsy carved pieces of perishable material. He also made great advancement in the treatment of decay and diseases of the teeth and gums, carrying the surgical treatment and preservation far beyond that understood by many others in the profession. He was among the first to adapt gold for filling teeth, in place of lead and tin, so generally used at that time. He was able to prepare and beat his own foil in the absence of gold beaters, and supplied other dentists with it.

Horace H. Hayden, of Windsor, Conn., who had been educated an architect, but was unsuccessful in that business, and having had occasion for the services of Dr. Greenwood, of New York, suddenly conceived the idea of trying his hand at the business. He settled in Baltimore in 1804, with little, if any instructions in dentistry. But, being an educated man, he at once saw the necessity of better understanding the profession which he had assumed, and of the collateral branches, that would aid him in his new position. He applied himself to the study of anatomy, physiology and surgery, and finally to general medicine.

His proficiency in these branches attracted the attention of the medical profession; and secured him recognition by the honorary degree of Doctor of Medicine, by both the University of Maryland and the Jefferson College of Philadelphia. In 1829, he delivered a course of lectures on dentistry to the medical class in the former institution; the first move in the direction of oral teaching in America. He was one of the founders of the Baltimore Dental College, and of the American Society of Surgeon Dentists, in 1830 and '40, and also one of the Editors of the Journal of Dental Science, in 1841. He died January 26, 1844, at the age of 75.

Edward Hudson had been thoroughly educated by his cousin and adopted father, a dentist of the highest position, as well as a man of letters, at Dublin, Ireland. He settled in Philadelphia in 1805, being a political refugee from England. Though Dr. Hudson is not credited with any writings relating to the profession of his choice; or of bringing out any particular discoveries, yet he enjoyed, in the highest degree, the reputation of being one of the first operators of his time; as well as a man of letters and high social worth. He died in 1833 at the age of 61.

Leonard Kœcker, an Englishman, came to New York in 1807; but soon removed to Philadelphia, where he remained in the practice of dentistry until 1822, when he returned to London. While in this country he published some valuable papers; and attained to a high reputation, especially as a dental surgeon; and afterwards added largely to English dental literature, through the London press. His treatise on "Conjoined suppuration of the Gums and Alveolus," published in the New York Medical Recorder, attracted considerable attention in medical as well as dental circles.

Jabez Parkhurst, born at Newark, New Jersey, October 4, 1764, after going through a series of occupations, or branches of business, finally settled on that of dentistry at the age of 43. He had the advantage of mature judgment, fine address and manners, and a classical education; which secured for him a high literary and social position. After qualifying himself as best he could, with such books as were then to be had, and attending lectures on anatomy, physiology and general surgery, he entered upon the practice, in New York in 1807. For twenty years he enjoyed the reputation of occupying the highest position in the ranks of the profession, with a large and lucrative practice. He died on the 29th of August, 1827.

Such is a brief sketch of some of the pioneers of the profession in this country. While I do not aim at a *history*, I mention these names with a few circumstances attending their connection with our calling, to show by what sort of men dentistry was nurtured in its infancy, and supported up to maturity; to demonstrate that it is well founded in science and true art; that it has always been recognized by professional men, and men of letters as a calling above that of an artizan; to show that, all through these years, the dark ages of dentistry, there have been men of education wielding a controlling power; who had a just appreciation of the qualification and needs of those entering the ranks.

The practice of this department of the healing art, having no legal restraint, and the public having but little ability to judge of real merit, many persons have been induced to take up dentistry as an easy business from which to make a living, though they were of doubtful honesty, as they possessed little or no knowledge of those sciences which it is so important for any person dealing with living tissues or human organs, to understand. Hence the numbers have multiplied with unprecedented rapidity.

At the commencement of this century, probably there were not more than ten dentists in the United States, and those were confined to the cities of Philadelphia, New York and Boston. In 1820 there were about 100; in 1830 about 300. From this time dentistry became more generally known, and the number increased more rapidly. In 1835 it was estimated that there were 600; in 1840 about 1,100; in 1850, 3,000; and now in 1870 there are not less than 10,000 persons in the United States calling themselves *dentists*.

Let us glance for a moment at the history of dental literature, and see if it has kept pace with the rapid growth in numbers.

Though the teeth, as distinguishing marks of beauty or deformity, in proportion to their perfection or imperfection, have been noted from the early history of our race; and there is mention made of their care and treatment, and the use of artificial substitutes in ancient times; yet we have no reliable account of dentistry as a separate branch of business until modern times. It has been reported that gold plugs have been discovered in the teeth of Egyptian mummies, but we have no history of the art of dentistry with that most educated and polished nation. What may have been known, what works on dentistry or records of the art may have been destroyed by the burning of their immense libraries at Alexandria, in the year 860, we shall never know. The pyramids or catacombs will never reveal them.

The dark centuries that followed developed nothing or even knew little if anything of the dental art. But in the revival of the arts and sciences in the sixteenth century, the teeth became a matter of interest to the learned, and treatment was instituted for their preservation or replacement.

Ambrose Pare, in his work on surgery, about the year 1560, not only spoke of the treatment of decayed and diseased teeth, but of the mode of making and inserting artificial ones, of bone or ivory, tied to the adjoining teeth with a thread, or gold or silver wire.

Eustachius published the first work exclusively devoted to the teeth, that we have any account of, in 1563, and Hemard, another, at Lyons in 1582. Benjamin Martin, nearly a century later, followed with a more elaborate work; but it was in the infancy of the art, with limited means for experiment or investigation, so that these writings attracted but little attention; but they were valuable, inasmuch as they inaugurated a series of papers that followed, on an almost unknown subject, thus stimulating investigation. Early in seventeen hundred, or in the beginning of the eighteenth century, the treatment of the teeth was regarded of so much importance in France that laws were promulgated regulating the practice of dentistry, requiring all who attempted it, to pass an examination by a board of surgeons or medical men. From the strict enforcement of this law but few persons became dentists, and those were principally educated surgeons. This is the first law relating to dentistry that we have any account of, and secured for the

French the just reputation, all through the eighteenth century, of being the best dentists, as well as the authorship of nearly all the dental literature in the world.

Fauchard, in 1728, published the first comprehensive treatise, with forty-two illustrations, reducing it to a regular system of theory and practice. It passed to its second edition, with improvements, in 1746. His sterling ability and achievements justly entitled him to the name of the "Father of Dentistry." His writings were the standard works for many years, and are still largely quoted.

From this time many small treatises or essays appeared in France; but Bourdet from 1754 to 1764, in a series of eight works, covered the whole ground so far as then known. He was followed by Jourdain, in 1761 and 1778, in a voluminous work in two volumes of 800 pages. Up to this time dental practice, as well as literature, was principally confined to the French, who were making rapid strides in developing and perfecting the art.

In 1771, John Hunter published in London, his work on "The Natural History, Formation, Growth, and Diseases of the Human Teeth," with illustrations; and in 1799 to 1801, Robert Blake, in Edinburg and Dublin, his "Essays upon the Structure of the Teeth in Man and various Animals," giving, as Fox says, the "first correct account of the manner in which the permanent teeth are formed."

In 1803 and 1806 Fox published his two famous works, "The Natural History and Diseases of the Human Teeth," and "The History and Diseases of the Teeth and Gums."

These three were the principal works emanating from the English press down to this time, and are still regarded as text books, though time and experience have detected many errors.

This brings us fully into the nineteenth century, with many valuable writings and able practitioners; but, as a specialty of the healing art, there was very much yet to be learned and written.

France had been the principal contributor, but from this time England rivalled if she did not surpass her in the character and extent of her publications. But nothing yet was produced from the American press, not a ray of dawning light from the region where the art of dentistry was so soon to be revolutionized, not one word from a people who were destined to take the foremost rank, as well in the conservative or surgical department as in the artificial.

Lead and tin were the principal materials used in filling decayed teeth; and bone and ivory, together with calves' and sheep's teeth, were used for artificial substitutes; or from the recommendations of Hunter and Fox, *transplanting* was largely practiced, deforming the peasantry and the poor, to beautify the nobility and the rich.

Porcelain teeth were unknown, though an attempt had been made at their manufacture in France as early as 1774, by M. Duchatean, an apothecary; but, having no knowledge of dentistry or of the characteristics or peculiarity of artificial teeth, so as to approximate natural ones in different individuals, he failed to attract attention. He, however, imparted his ideas to Dubois de Chemant, a dentist of position, who so improved upon them, that Louis XVI granted him the first patent, so far as known, in dentistry. He wrote several small works on their manufacture and adaptability, as "incorruptible" teeth. His claim as the inventor of porcelain teeth has always been acknowledged, though no very great degree of perfection had been reached.

Delabarre materially improved upon Chemant's work, and Audibran still farther advanced in the right direction, and gave to the world, in 1821, a well written volume, giving his formulas with full instructions, and introduced their use to a considerable extent in France. But it was for American skill and enterprise to carry their manufacture to a degree of perfection, so as to utterly supplant those of other materials. This was due, in a great measure, to James Alcock, of New York and Samuel W. Stockton, of Philadelphia, commencing about the year 1831–2, but not reaching a very high degree of perfection until 1835–6. Their formulas have been improved, and their experience turned to so good an account, that now, nearly all the porcelain teeth used in the world are of American manufacture.

We find nothing emanating from American dentists to enrich the shelves of dental libraries, or to add to dental literature, prior to 1819, when Kœcker's article appeared in the New York Medical Recorder, on "Conjoined Suppuration of the Gums and Alveolus"; and Dr. L. S. Parmly printed, for the use of his patients, a pamphlet called "A Practical Guide to the Management of the Teeth"; and in 1821 another similar work on "The Natural History and Management of the Teeth."

In 1822 Eleazer Parmly published a small volume on the

"Diseases and Treatment of the teeth"; and in the same year, Dr. J. F. Flagg, of Boston, a similar work, called "A Treatise on the Structure, Formation, Disease and Treatment of the Human Teeth"; John Trenor, of New York, in 1824 published a paper in the Medical Recorder on "Tic Douloureux;" and in 1826 another on the "Stucture, Organization and Nourishment of the Teeth." In 1827, Gardette published his only article in the same paper, on "Transplantation of the Human Teeth", pointing out the dangers and fallacy of the practice so inhuman and cruel.

In 1829 Samuel S. Fitch published a "System of Dental Surgery," a book of 500 pages, which passed to its second edition in 1835, much enlarged, improved and illustrated. This was the most complete and comprehensive work, covering the general principles and practice of dentistry, published up to this time, and was for many years the standard text-book.

In 1839 Chapin A. Harris produced his first work under the name of "Dental Art.—a practical Treatise on Dental Surgery," which, with many corrections and additions, and after having gone through several editions, is now known as "Harris' Principles and Practice of Dental Surgery," and is doubtless familiar to us all.

In the year 1839 or 1840, was the turning point, in America, in Dental literature; as well as in the character and tone of the profession generally. Many able pens have been at work since that time, and greatly multiplied books, on several departments of the science and art; but there is still much more needed.

In the matter of Dental journalism, there has been as rapid a growth, as in book making. "The American Journal of Dental Science" was started in December, 1840, by Chapin A. Harris and Eleazer Parmly, as the pioneer of this now numerous class of publications, that carry to the office and the laboratory a constant stream of practical knowledge that can not be found in any standard text book.

Thus dentistry was rapidly advancing to the rank of the learned professions, without an educational institution or a department in any of the halls of learning, where it could be taught.

Though it was at a great pecuniary risk, yet, so urgent was the necessity, a charter was procured of the Legislature of Maryland; and with Harris, Hayden, Bond, and a few others more ambitious for professional advancement than for money, the "Baltimore College of Dental Surgery" was started in 1840; which for awhile stood alone like a beacon light to the way-farer. In 1846 the "Ohio College of Dental Surgery" was established in Cincinnati, which gave a Western as well as an Eastern guiding star. These two schools, though at a pecuniary loss to the faculty, demonstrated not only the practicability of teaching dentistry by lectures and a regular collegiate course, but the necessity of such institutions. In 1856, Philadelphia, the emporium of medical education in America, moved in this direction. Thus this department of Dental progress has advanced, until to-day we can boast ten regularly organized Dental Colleges and University departments in the United States.

The next, though by no means the least step in advancement, was the organization and support of Dental Societies; which have been the moving instrumentality in the production of much of the written science of dentistry, the establishment of Dental Colleges, and the advancement of the standard of the profession to its present high position in this country. They are the best channels for obtaining a correct practical knowledge, and enable one to keep pace with the rapid progress now being made.

The first organization that I know of was that of the "American Society of Dental Surgeons, in 1840; then the "Mississippi Valley Dental Association," in 1844, embracing the country West of the Alleghany Ridge. The former society, as the pioneer, took a very high position, suited to the tastes of scientific and professional men, somewhat in advance of the state of the profession at that time; as systems and modes of practice had not yet settled down to an absolute standard. There was too much exclusiveness and conflict of opinion. In fact the *mercurial taint* so fired up the spirit of their investigations, that the Society was stranded on the shoals of *amalgam* in 1854, and the ship soon after went to pieces; but it had filled an important mission.

There was a just demand for a society on a more liberal basis, which resulted in the reorganization of the "American Dental Convention" in 1855, which with open doors and outstretched arms, still welcomes to annual membership any Dentist who wishes to attend. It has done a great work in the advancement of practical knowledge, steering clear of all factions and specialties. From an ambition for a higher tone of associated investigation in the various departments of Dental science, a few persons as delegates from such local societies as then existed, formed themselves into the "American Dental Association," at Niagara Falls, in 1859, which was to be a delegated body, made up of the representative men of the profession. This is our present National Society, that we are all acquainted with; and we hope it may long exist as the pride and honor of the profession. Since its organization, local societies have sprung up in cities and districts all over the country, and have thus augmented the membership of that Association, as well as securing great benefit and improvement at home.

Of all these, the "Dental Society of the State of New York," with its eight districts, are the first that have been organized under any law relating to Dentistry, or in any way recognizing it as a profession. The Empire State has taken the lead, passing the first Dental law in the United States.

We are now thoroughly established in the several society organizations under that law, and must go on thoroughly at work in advancing the *science*, as well as the *art*, and carry the profession in this State to an elevated position. This law virtually consigns to the profession itself the right and power to pass judgment upon all those who are hereafter to be admitted into its ranks. This is as it should be. An able reviewer says, "The history of all the learned professions proves that none of them become extensively useful or respectable, except under the immediate restraint of their own membership." It is the well informed Dentists who are the best judges as to the qualification and fitness of those who are to exercise the functions of the Dentist; and "unless we maintain, at least, the level of our well educated classes, the dignity of the professional man is lost in the character of the tradesman; thus dragging down, as far as an individual can do it, the professional reputation of the whole body. Whatever is a man's pursuit in life, it is knowledge and moral character that gives him his real rank and position."

For the advancement of any branch of business, and particularly a profession, made up in part at least of theories, the truth or falsity of which must be proven by practical application, there must be concert of action, interchange of opinions, and comparison of the results from actual practice. No science or profession ever became established as such by single-handed individual labor. Individuals have made important scientific discoveries and progress in the arts; but it can not be a revealed science or a profession until in the hands of associations of men, who jointly and severally examine and experiment, until theories prove to be fixed facts and living principles. Then a system may be in truth a *science*, and the knowledge of that system, reduced to practice, a *profession*. An old proverb says, "one man is no man," which really illustrates the impotency of an individual, shut out from the aid and councils of his fellows; striving as a man groping in the dark for knowledge; but at the same time declining to avail himself of the surest means of obtaining it. He wastes more time and more energy in obtaining very imperfect information, than would secure a high degree of attainment, by a free and full intercourse with those of his own calling.

Never in the history of the world have there been so many avenues opened, so many facilities offered for obtaining knowledge in all the various branches of science and art, or business and letters, as to-day. Only a generation back, or at a period when some of us entered upon the practice of dentistry, there was very little opportunity for obtaining even a moderate knowledge of the art. We were obliged to plod along, as best we could, and dig out as our ingenuity or observation might suggest; or carry into practice a hint that might casually escape or be wrung out of some of the older operators. There were no Dental colleges or associations, nor very little concert of action; no Dental journals and very little Dental literature in this country. But now the whole condition of things has changed. We have Dental periodical literature, and standard text books; Dental colleges with a regular curriculum of study; Dental associations and a free interchange of opinions and practice. All these are placed within the reach of every person, so that there is no longer any excuse for obtruding upon the public unskillful and incompetent Dentists. And now the legislature extends to us not only recognition as a profession, but the arm of legal support, and an honorable legal title. Though we have not yet reached the acme of our ambition, is not this *progress*, and that too at a pace that should encourage and console if not fully satisfy us? It is a delightful foreshadowing of the great future of dentistry.

